# Quantifying the supply-demand relationship of ecosystem services to identify ecological management zoning: A case study in mountainous areas of northwest Yunnan, China

**DOI:** 10.1016/j.heliyon.2024.e32006

**Published:** 2024-05-28

**Authors:** Xiaobao Wang, Xiaoqing Zhao, Yifei Xu, Yuju Ran, Xianmin Ye, Yuqin Zhou, Beihao Wu, Bocheng Chu

**Affiliations:** a School of Earth Sciences, Yunnan University, Kunming, 650500, China; b Institute of International Rivers and Eco-Security, Yunnan University, Kunming, 650500, China; c Yunnan Fenglan Project Investment Consulting Group Co., Ltd., Kunming, 650500, China

**Keywords:** Ecosystem services, Supply-demand relationship, Coupling coordination degree, Ecological management zoning, Mountainous areas of northwest Yunnan

## Abstract

Establishing ecological management zones based on the supply-demand relationship of ecosystem services (ESs) is essential for fostering sustainable development within social-ecological systems and improving human well-being. In this study, the spatial pattern between supply and demand in five ESs (grain production (GP), carbon sequestration (CS), soil conservation (SC), water conservation (WC), and habitat quality (HQ)) is analyzed using the ESs supply-demand ratio (ESDR) method, the spatial autocorrelation method, and the coupled coordination degree model. Zoning is performed according to the differences in their spatial combinations, and differential zoning management policies are proposed. The following results were obtained: (1) In terms of the ESDR, except for a slight increase in GP surplus from 2010 to 2020, there is a decline in the surplus of the other four ESs. (2) CS, WC, and HQ are dominated by cluster types LH and HL. GP and SC are dominated by cluster types HH and LL. The average value of the coupling coordination degree (CCD) of comprehensive ESs supply and demand show five types: moderate disharmony, slight disharmony, near disharmony, basic coordination, and slight coordination. (3) Based on the multiple spatial heterogeneity of ESs supply and demand, differentiated ecological management strategies are proposed at the grid scale. Overall, this study discover the spatial pattern of mismatch between the supply and demand of ecosystem services (ESs) in mountainous urban areas. This contribution enhances the discourse surrounding sustainable development theory and advances research on the coupling of social-ecological systems. Furthermore, it offers valuable insights for the formulation of sustainable ecological management policies tailored to mountainous urban settings.

## 1 Introduction

ESs encompass the benefits that humans derive directly or indirectly from ecosystems [1]. These services form the foundation for human survival and development [2] and serve as the bridge linking the natural environment and social economy systems [3]. ESs supply reflects the capacity of ecosystems to provide products and services to humans [4]. Furthermore, ESs demand is perceived as the total of all ecosystem goods and services consumed or utilized within a specific region and period [5,6]. The ESs supply and demand constitute the dynamic process of ESs transitioning from natural ecosystems to human social systems [7]. With industrialization and urbanization, unreasonable human activities cause ecological and environmental issues and ecological space reduction [8]. Ecosystems are encountering increasingly prevalent threats and challenges, which undermine the capacity of ESs supply. Concurrently, social economy development is driving a continuous increase in human demand for various ESs, aggravating the imbalance between the supply and demand of regional ESs and environmental degradation. It directly or indirectly restricts the sustainable development of the social economy. Consequently, clarifying the relationship between ESs supply and demand and analyzing their spatial differentiation and agglomeration characteristics are essential to achieving sustainable development of social-ecological system and optimal allocation of natural resources [9].

ESs supply and demand have become a hot topic in global ESs research, which is indispensable in promoting sustainable ecological development and improving human well-being. Research in this field, focusing on ecological carrying capacity and ecosystem service value [10], commenced in the 1990s [1]. Previous studies on the supply and demand of ESs mainly focused on ESs supply [11], involving its concepts, classifications, and quantitative methods [1,[12], [13], [14]]. In recent years, the research on the relationship between ESs supply and demand has been paid more and more attention. Yuan et al. (2023) [15] analyzed the trade-offs and synergies between ESs supply and demand. Wei et al. (2023) [16] examined the agglomeration characteristics of the supply and demand of ESs, using cold and hot spots analysis and local spatial autocorrelation. Many scholars also studied the spatial mismatch between supply and demand [[17], [18], [19], [20], [21]]. These studies visualize the spatial relationship between the supply and demand of ESs and reveal the difference between supply and demand, providing support for ecological management zoning based on the ESs supply-demand relationship. However, the time scale of ecological management zoning research is primarily static, ignoring the complexity, dynamics, and non-linear correlation of the ESs supply and demand. It also lacks a comprehensive spatiotemporal dynamic analysis of the relationship between the ESs supply and demand [22,23]. At the same time, ecological management zoning research treats the supply and demand of ESs as two independent variables, and few studies consider their correlation [24]. Existing research seldom considers ecological management zoning comprehensively from the perspective of the supply and demand of ESs.

Research on ecological management zoning predicated on ESs is relatively extensive. Zeng et al. (2023) [25] employ local spatial autocorrelation to compute the agglomeration type of ESs supply and demand for zoning. Huang et al. (2023) [26] utilize the K-means clustering method for zoning. Many scholars have undertaken ecological management zoning from various landscape perspectives, including water [27], farmland [9], wetlands [28], and typical urban areas [29]. Ecological management zoning also encompasses zones from the perspective of the ESs cluster [30,31], ecological restoration zoning [32], ecological compensation zoning [16]. Some studies have revealed the response relationship between the balance of ESs and the proportion of land use types. Ecological management can be carried out by controlling the proportion of land use, thereby promoting the balance of ESs [33,34]. In summary, research on ecological management zoning is progressively maturing, enriching the understanding of ESs. It has become an essential basis for ecological protection and ecological space control [29]. However, few studies concerning the spatial coupling between the supply and demand of ESs, and few scholars have explored ecological management zoning based on the degree of coupling between ESs supply and demand. Ecological zoning based on the coupling and coordination relationship between ESs supply and demand can reflect the degree of coordinated development and ecological sustainability of the man-land systems in space and provide valuable references for ecological protection and ecological management [35].

Consequently, a key research question revolves around promoting the balance between the supply and demand of ESs to achieve regional sustainable development through the differentiated management of territorial space. Based on previous research, this study identified multiple ESs supply and demand relationships, develops a multi-level zoning management framework, and proposed management strategies to promote ESs supply-demand balance. The objectives of this study includes: (1) assessing the spatial and temporal variations in supply, demand, and supply-demand relationship of ESs; (2) analyzing the cluster characteristics and the coordination degree between the ESs supply and demand; (3) establishing ecological management zones, and proposing corresponding management measures to provide scientific references for managing ESs and territorial space.

## 2 Materials and methods

### 2.1 Study area

**Fig. 1:**
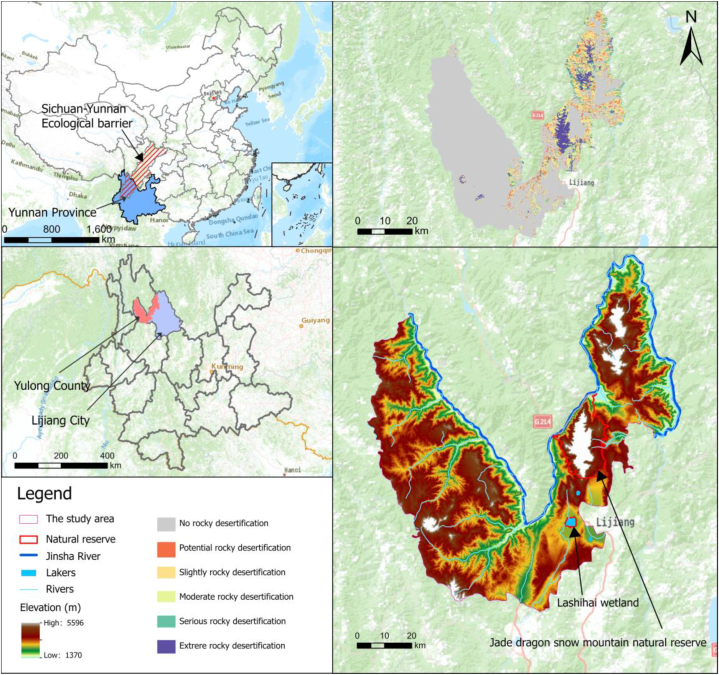
The location of the study area.

Yulong County (99°23′–100°32′E, 26°34′–27°46′N) is located in Lijiang City, northwest of Yunnan Province, covering a total area of the county is 6198.76 km^2^ (Fig. 1). The landform belongs to the alpine gorge area of the Hengduan Mountains, the highest altitude is 5596 m, and the topography landforms complex. The county's forest coverage rate was 76.85 % at the end of 2020, and the proportion of ecological protection red line area was 47.1 %. It is the habitat of the national key protected animals and plants, the ecological barrier area of Sichuan and Yunnan, and the key forest area of Yunnan Province. Yulong County boasts a wealth of tourism resources, such as the Jade Dragon Snow Mountain, Tiger Jumping Gorge, and Blue Moon Valley. Meanwhile, Yulong County also grapples with serious issues of rocky desertification. The karst landform area accounts for 30.85 % of the country's area. With the rapid economic development, continuous urbanization, and intensification of tourism activities, the conflict between ecological protection and socio-economic development needs has become increasingly prominent in Yulong County. Therefore, the mismatch between the ESs supply and demand tends to intensify, posing a severe threat to regional sustainable development.

### 2.2 Data sources

We converted all spatial data into the WGS_1984_UTM_47 N projection system to ensure the consistency of spatial coordinates. In this study, we utilized 1 km*1 km grids as the spatial unit and divided Yulong County into 6601 grids using fishing nets in ArcGIS 10.2. Specific data information is shown in Table 1.

**Table 1:** Main data and sources.

Data types	Sources	Formats
Landsat-4/5TM images	http://www.gscloud.cn/	Raster
Sentinel-2B images	https://scihub.copernicus.eu/	Raster
Net primary productivity	https://lpdaac.usgs.gov/	Raster
Digital elevation model	http://www.gscloud.cn/	Raster
Soil properties	https://www.geodata.cn/	Raster
Meteorological data	https://www.geodata.cn/	Vector/table
Population distribution	https://www.geodata.cn/	Raster
Statistical data	Statistical yearbook and statistical bulletin	Table/text
Fundamental geographic information	https://www.webmap.cn/	Vector

**Fig. 2:**
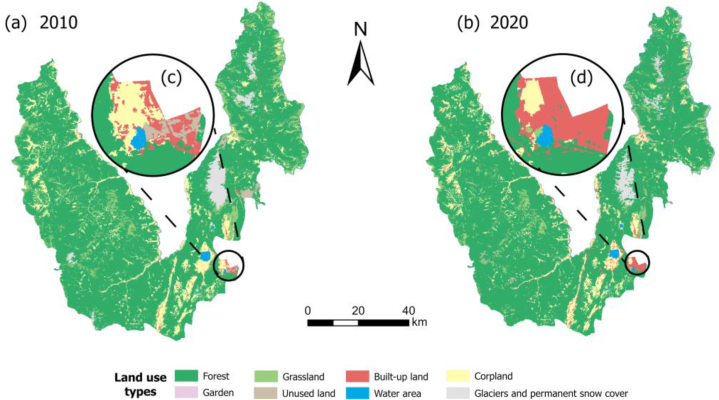
Land use spatial distribution. (a) Land use in 2010; (b) Land use in 2020; (c) Land use thumbnail in 2010; (d) Land use thumbnail in 2020.

Using Landsat-4/5TM (resolution: 30 m × 30 m) and Sentinel-2B images (resolution: 10 m × 10 m), the land use types in 2010 and 2020 were extracted by human-computer interaction interpretation. We divided eight land use types with a unified resolution of 30 m × 30 m (Fig. 2). The overall accuracy of the interpretation results in 2010 and 2020 was 93.3 % and 95.1 %, and the average Kappa coefficients of the samples were 0.88 and 0.91, which met the requirements of the study.

### 2.3 Quantification of ESs supply and demand

#### 2.3.1 Grain production

##### 2.3.1.1 Supply

GP is an important guarantee for the survival and development of residents and is of great significance for maintaining regional security. Studies have shown a significant linear relationship between crop and livestock production and normalized difference vegetation index (NDVI) [36]. Based on the land use types, the total GP in the statistical yearbook was allocated according to the proportion of each pixel's VCI to the total VCI in cropland to obtain GP supply [37].

We used grain consumption to represent GP demand, mainly including grains, potatoes, and beans, among which the demand of urban and rural populations is quite different [37]. The Yulong Statistical Yearbook showed that the urban population's annual per capita grain consumption was 112.87 and 104.68 kg in 2010 and 2020, and that of the rural population was 158.02 and 146.66 kg. We allocated grain production demand to each spatial pixel based on the revised population distribution data. The formula is as follows:

(1) $$GP\left(x\right)=GP_{t}\times \frac{VCI\left(x\right)}{\sum_{x=1}^{n}VCI\left(x\right)}$$

(2) $$VCI_{i}\left(x\right)=\left(\frac{NDVI_{i}\left(x\right)-NDVI_{i}\left(\min\right)}{NDVI_{i}\left(\max\right)-NDVI_{i}\left(\min\right)}\right)\times 100\%$$

Where *GP (x)* is the grain production supply of pixel *x*; *GP*_*t*_ is the total grain output in the whole region; n is the total pixel number of cropland; *NDVI(x)* is the *NDVI* of the pixel *x* in cropland; *NDVI*_*max*_ and *NDVI*_*min*_ represent the maximum and minimum of *NDVI* in cropland, respectively. *VCI(x)* is the grain production demand of pixel x;

##### 2.3.1.2 demand

We used grain consumption to represent GP demand, mainly including grains, potatoes, and beans, among which the demand of urban and rural populations is quite different [37]. The Yulong Statistical Yearbook showed that the urban population's annual per capita grain consumption was 112.87 and 104.68 kg in 2010 and 2020, and that of the rural population was 158.02 and 146.66 kg. We allocated GP demand to each spatial pixel based on the revised population distribution data. The formula is as follows:

(3) GPD=ρ(x)*per

Where *ρ(x)* is the population density of pixel *x*, per is the annual per capita grain consumption, and the standards of urban and rural populations are different.

#### 2.3.2 Carbon sequestration

##### 2.3.2.1 Supply

CS is the ability of ecosystems to capture carbon from the atmosphere through their functions and growth processes over a given period. Plant biomass can reflect the atmospheric CO_2_ sequestration in terrestrial ecosystems. We calculated the carbon sequestration supply based on vegetation's photosynthesis and respiration equations [38]. The formula is as follows:

(4) $$Q_{{\text{CO}}_{2}}=M_{{\text{CO}}_{2}}/M_{C}\times NEP$$

(5) $$NEP=\alpha *NPP*M_{C_{6}}/M_{C_{6}H_{10}O_{5}}$$

Where $Q_{{\text{CO}}_{2}}$ do ecosystems sequester the amount of *CO*_*2*_ (*t·CO2/a*); $M_{{\text{CO}}_{2}}/M_{C}$ is the coefficient of converting *C* into *CO*_*2*_, i.e., 44/12; *NEP* is the net ecosystem productivity (*t·C/a*). *α* is the *NEP* and *NPP* conversion coefficient; *NPP* is net primary productivity (*t·dry matter/a*); $M_{C_{6}}/M_{C_{6}H_{10}O_{5}}$ is the coefficient of dry matter conversion to *C*, namely 72/162.

##### 2.3.2.2 Demand

Carbon emissions express the demand for carbon sinks. According to the Yulong Statistical Yearbook, we calculate the carbon emissions of energy consumption and the carbon emissions of agricultural production. The former is allocated to each spatial pixel according to population density, and the latter is equally allocated to paddy fields and dry land [39]. The formula is as follows:

(6) $$E_{engry\;consumption\;}\left(x\right)=E_{GDP}\left(x\right)\times GDP\left(x\right)\times 0.7172$$

(7) $$E_{paddy\;field}\left(x\right)=\delta_{m}\times 12/16+\sum_{j=1}^{n}T_{j}\left(x\right)+\sigma_{j}\left(x\right)$$

(8) $$E_{dry\;field}\left(x\right)=\sum_{j=1}^{n}T_{j}\left(x\right)+\sigma_{j}\left(x\right)$$

Where *E*_*nergy consumption*_ *(x)* is the carbon emissions by energy consumption at the pixel x(t); E_GDP_ is the unit GDP energy consumption at the pixel *x* (t standard coal/10,000 CNY); GDP is the total GDP at the pixel *x* (10,000 CNY); 0.7172 is the revised carbon emission coefficient. *E*_*paddy field*_*(x)* is the carbon emission of pixel x in paddy field; *δ*_*m*_ is the carbon emission coefficient of CH_4_ in a single rice paddy field, that is, 156.2 kg/hm^2^; 12/16 is the coefficient of CH_4_ converting into C; *T*_*j*_*(x)* is the total carbon emission of the j-th influencing factor at the pixel x, *σ*_*j*_*(x)* is the carbon emission parameter of each influencing factor at the pixel x. *E*_*dry field*_ *(x)* is the carbon emission of pixel *x* in dry land.

#### 2.3.3 Soil retention

##### 2.3.3.1 Supply

SR is conducive to reducing soil erosion and restoring soil fertility. We used the modified universal soil loss equation (RUSLE) to estimate regional soil conservation and erosion [40,41]. Soil retention was used as the supply of soil retention. The formula is as follows:

(9) SRS=R×K×LS×(1−C×P)

Where *R* is the rainfall erosion factor, *K* is the soil erosion factor, *LS* is the factor of slope length and slope gradient, severally; *P* is the soil conservation measures factor; *C* is the vegetation cover factor;

##### 2.3.3.2 demand

According to the study of Villamagna et al. (2013) [42], the severity of soil erosion indicates to what extent the land or ecosystem should be restored. The amount of erosion expected by humans is taken as the demand for SR services. The formula is as follows:

(10) SRD=R×K×LS×C×P

where the meanings of all parameters here are consistent with those in formula (9).

#### 2.3.4 Water conservation

##### 2.3.4.1 Supply

We define water conservation as the capacity of ecosystems to capture or store water from rainfall and simultaneously minimize surface runoff. In this study, the InVEST model was used to obtain the supply of water conservation in Yulong County [43]. The formula is as follows:

(11) $$WY\left(xj\right)=\left(1-\frac{AET\left(xj\right)}{P\left(x\right)}\right)\cdot P\left(x\right)$$

Where *WY(xj)* is the water yield of pixel x in land use type j; *AET(xj)* is the actual evapotranspiration of pixel x in land use type j; *P(x)* is the precipitation of the pixel x.

##### 2.3.4.2 Demand

Water conservation demand is expressed in terms of water consumption [44], the agricultural and industrial use level is equally allocated to farmland, industrial, and mining land, and the ecological level is equally allocated to the land and waters of the built-up area; domestic water is allocated to pixels according to population density [45]. The formula is as follows:

(12) $$D_{WC}=D_{\text{agricultural}\;}+D_{\text{industrial}\;}+D_{\text{living}\;}+D_{\text{ecological}\;}$$

Where *D*_*WC*_ is water demand, *D*_*agricultural*_, *D*_*industrial*_, *D*_*living*_, and *D*_*ecologica*l_ represent agricultural, industrial, living, and ecological water demand, respectively.

#### 2.3.5 Habitat quality

##### 2.3.5.1 Supply

The InVEST model is used to evaluate the supply of HQ [46]. Specifically, the habitat degradation degree was calculated by combining the sensitivity of different land use types to threat sources and the intensity of external threats, and the habitat quality was further calculated. The formula is as follows:

(13) $$Q\left(xj\right)=H\left(j\right)\left(1-\frac{D^{z}\left(xj\right)}{D^{z}\left(xj\right)+k^{z}}\right)$$

Where *Q(xj)* refers to the hq supply of pixel x in land use type *j*; *H(j)* is the habitat adaptability of land use type *j*; *D(xj)* is the habitat degradation degree of pixel x in land-use type *j*; *k* is the half-saturation constant; *z* is the normalized constant, usually 2.5.

##### 2.3.5.2 Demand

We defined each grid's habitat quality demand (HQD) as the difference between the demand standard and the grid habitat quality supply. The formula is as follows:

(14) $$HQD_{st}=\frac{\sum_{k=1}^{M}Q\left(x\right)}{M}$$

(15) $$HQD\left(x\right)=\left\{ \begin{array}{cc} HQD_{st}-Q\left(x\right), & Q\left(x\right)<HQD_{st}\\ 0 & Q\left(x\right)⩾HQD_{st} \end{array}\right.$$

Where *HQD(x)* is the habitat quality demand of pixel x; *HQD*_*st*_ is the habitat quality demand standard; *Q(x)* is the habitat quality supply of pixel x; *M* is the total count of pixels in the whole region.

### 2.4 Quantification of the ESDR

The ESDR represents the relative magnitude of supply and demand for ESs. It visualizes the spatial consistency and matching between the supply and demand of each ESs [47]. Therefore, this study promotes ESDR to express the relationship between the supply and demand of ESs. The formula is as follows:

(16) $$ESDR_{ij}=\frac{ES_{ij}^{S}-ES_{ij}^{D}}{\left(ES_{i}^{S\;\max}+ES_{i}^{D\;\max}\right)/2},\left\{ \begin{array}{c} \geq 0，\text{surplus}\\ ＜0，\text{deficit} \end{array}\right.$$

Where *ESDR*_*i*j_ is the ecological supply-demand ratio for ESs type i in grid j, $ES_{ij}^{S}$ the supply of ESs type i in grid j, $ES_{ij}^{D}$ the demand of ESs type i in grid j, $ES_{i}^{S\;\max}$ the maximum supply of ESs type i, $ES_{i}^{D\;\max}$ and the maximum demand of ESs type i.

### 2.5 Quantification of the comprehensive ecological supply and demand ratio

The comprehensive ecological supply and demand ratio (CESDR), calculated as the arithmetic mean of the ESDR, is used to evaluate the ESs at an integrated level [48]. The natural breakpoint method divides the CESDR into three levels to classify the different levels of the CESDR. The calculation formula is as follows:

(17) $$CESDR_{j}=\frac{1}{n}\sum_{i=1}^{n}ESDR_{ij},\left\{ \begin{array}{cc} >0.1, & \text{surplus}\;\\ \in \left[-0.1,0.1\right], & \;\text{near}\;\text{balance}\;\\ <-0.1, & \text{deficit}\; \end{array}\right.$$

Where CESDR is the comprehensive ESs supply and demand ratio, *CESDR*_*j*_ is the CESDR in grid j, n is the number of ESs types, and *ESDR*_*i*j_ is the ESDR of ESs type i in grid j.

### 2.6 Identifying spatiotemporal matching of the ESs supply and demand

We conducted spatial autocorrelation analysis of the multiple ESs supply and demand using the global and local autocorrelation Moran's *I* index by Geoda. Global spatial autocorrelation is used to study the overall spatial relationship, and local spatial autocorrelation is used to study spatial variation status [49].

#### 2.6.1 Global spatial autocorrelation

Global spatial autocorrelation is analyzed by global Moran's *I* [11]. The formula is as follows:

(18) $$I=\frac{\sum_{i=1}^{n}\sum_{j=1}^{n}w_{ij}\left(x_{i}-\bar{x}\right)\left(x_{j}-\bar{x}\right)}{\sum_{i=1}^{n}\sum_{j=1}^{n}w_{ij}{\left(x_{i}-\bar{x}\right)}^{2}}$$

Where *x*_*i*_ is the attribute value of the spatial grid i, $\bar{x}$ is the mean value of the attribute; *n* is the total number of spatial grids; *w*_*ij*_ is the spatial weight matrix between the spatial grid i and grid j.

#### 2.6.2 Local spatial autocorrelation

The bivariate local spatial autocorrelation can directly reflect the correlation between the supply and demand of ESs and the spatial agglomeration between adjacent spatial units [50]. The cluster type distinguishes between a statistically significant cluster of low values (LL), the cluster of high values (HH), outliers with a low value surrounded primarily by high values (LH), and outliers with a high value surrounded primarily by low values (HL). The formula is as follows:

(19) $$I_{i}=\frac{x_{i}-\bar{x}}{n\sum_{i}{\left(x_{i}-\bar{x}\right)}^{2}}\sum_{j}w_{ij}\left(x_{i}-\bar{x}\right)$$

where the meanings of all parameters here are consistent with those in formula (18).

### 2.7 Calculation of CCD between ESs supply and demand

CCD describes the degree of interaction and coordination between the systems [51,52]. This model measures the coupling relationship between the supply and demand of ESs [16]. The formula is as follows:

(20) $$D=\sqrt{C\times T}$$

(21) $$C=2\times \sqrt{\frac{X_{S}\times X_{D}}{{\left(X_{S}+X_{D}\right)}^{2}}}$$

(22) $$T=\alpha \times X_{S}+\beta \times X_{D}$$

Where *D* is the coupling coordination degree, *D* ∈ [0, 1], the greater the *D*, the better the coordination of ESs supply and demand; *C* is the coupling degree; *T* is the comprehensive coordination index; *X*_*S*_ indicates the ES supply; *X*_*D*_ indicates the ESs demand; *α*, *β* are the weight coefficients, the importance of ESs supply and demand is the same, so take *α* = *β* = 0.5 [51].

Referring to relevant research results [53], according to the size of the coupling coordination degree value, the CCD of comprehensive ESs supply and demand is divided into different types.

(23) $$D=\left\{ \begin{array}{l} \;\left[0.00,0.10\right)\;\;\;Extreme\;disharmony\\ \;\left[0.10,0.20\right)\;Severe\;disharmony\\ \;\left[0.20,0.30\right)\;Moderate\;disharmony\\ \;\left[0.30,0.40\right)\;\;\;\;\;Slight\;disharmony\\ \;\left[0.40,0.50\right)\;\;\;\;\;\;Near\;disharmony\\ \;\left[0.50,0.60\right)\;\;\;\;Basic\;coordination\\ \;\left[0.60,0.70\right)\;\;\;Slight\;coordination\\ \;\left[0.70,0.80\right)\;\;Moderate\;coordination\\ \;\left[0.80,0.90\right)\;\;\;Good\;coordination\\ \;\left[0.90,1.00\right)\;Quality\;coordination \end{array}\right.$$

### 2.8 Ecological management zoning

Considering the spatial combination of the comprehensive supply-demand ratio, coupling coordination degree, and agglomeration characteristics, we divided Yulong County into primary ecological zones based on the unbalanced ESs supply and demand characteristics. Further, the secondary ecological zones are subdivided, considering the spatial coupling characteristics and the agglomeration characteristics of ESs.

## 3 Results

### 3.1 Spatial-temporal characteristics of multiple ESDR

#### 3.1.1 Characteristics of ESs supply and demand

This study reveals a mismatch between the supply and demand of five ESs types. The supply of five ESs surpasses demand, indicating ESDR is in surplus. However, judging from the changes in the gap between ESs supply and demand, the surplus of GP increased from 111.36 kg/hm^2^ in 2010 to 124.86 kg/hm^2^ in 2020, while the surplus of the other four ESs decreased. Specifically, CS, SC, WC, and HQ decreased by 1.136 t/hm^2^, 10.47 t/hm^2^, 13.51 mm, and 0.003 (Table 2).

**Table 2:** The average supply and demand of ESs from 2010 to 2020.

ES types (units)	Support		Demand		Supply minus demand	
	2010	2020	2010	2020	2010	2020
Grain production (kg/hm^2^)	165.7	176.98	54.34	52.12	111.36	124.86
Carbon sequestration (t/hm^2^)	2.936	2.971	1.563	2.734	1.373	0.237
Soil conservation (t/hm^2^)	305.69	298.39	3.71	6.88	301.98	291.51
Water conservation (mm)	65.12	54.9	20.51	23.8	44.61	31.1
Habitat quality	0.875	0.872	0.0861	0.0863	0.789	0.786

#### 3.1.2 Spatial distribution of the ESDR

**Fig. 3:**
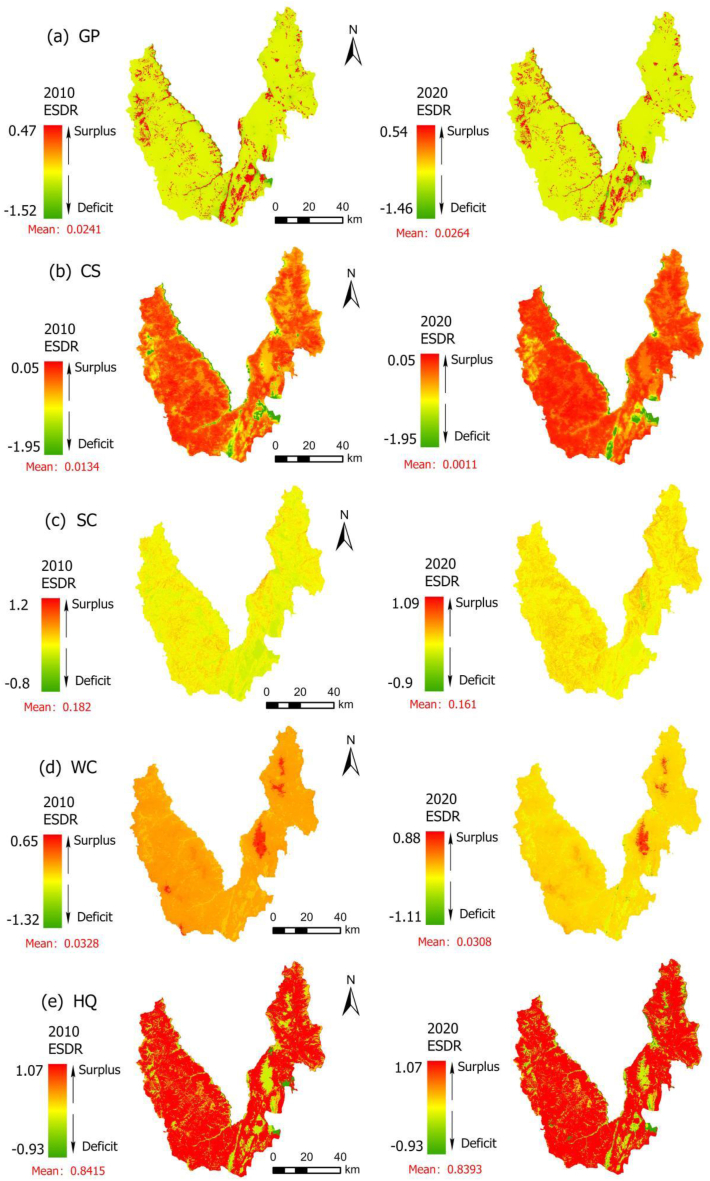
2010–2020 Spatial distribution of the ESDR. (a) GP; (b) CS; (c) SC; (d) WC (e) HQ.

The proportion of surplus areas for GP has experienced a decline, decreasing from 9.6 % to 8.89 % (Fig. 3-a). These areas are predominantly concentrated in the Jinsha River Valley and the adjacent flat basin. Notably, the mean ESDR has increased from 0.0241 to 0.0264, signifying an overall expansion of the region's ecological services surplus.

The surplus area of CS constitutes a substantial proportion; however, its spatial extent has gradually decreased from 84.90 % to 76.96 % (Fig. 3-b). These surplus areas are predominantly concentrated in mountainous regions characterized by high vegetation coverage. Simultaneously, the mean ESDR significantly declined from 0.0134 to 0.0011, indicating a decrease in the overall ESs surplus across the region. Conversely, the deficit area of CS is localized in specific zones: the coastal region along the Jinsha River, the urban built-up areas in the southeast, and the mountain basin regions in both east and west. Notably, this deficit area has expanded, with its proportion increasing from 15.10 % to 23.04 %.

The surplus area of SR accounted for an absolute advantage because the mountainous areas of Yulong County have high vegetation coverage, which strengthens the effect of plant roots on soil, effectively reduces soil erosion, and promotes soil conservation. However, the overall surplus decreased slightly, from 99.97 % to 99.70 %, and the mean ESDR decreased from 0.182 to 0.161 (Fig. 3-c). The area with a deficit in the SR primarily resides in the Jade Dragon Snow Mountain Scenic Area, and its proportion has increased from 0.03 % to 0.30 %. The primary reason is that the area is a high-altitude rocky desertification region, and the process of rocky desertification accelerates soil degradation. Furthermore, human activities, such as tourism and infrastructure development, impact SR to a deficit.

The WC was predominantly in the surplus area, with the proportion experiencing a slight increase from 89.38 % to 89.79 % (Fig. 3-d). The high surplus area of WC is mainly concentrated in the Jade Dragon Snow Mountain Scenic Area, which is covered by glaciers and permanent snow and stores a large amount of water resources. Conversely, deficit regions for WC are primarily concentrated in specific zones: the flat basin, the eastern and western mountain basins, and the Jinsha River valley. These areas exhibit higher cropland density, larger populations, and substantial water resource demands, resulting in a proportion decrease from 10.62 % to 10.21 %.

The surplus area of HQ was dominant, yet the surplus area decreased from 98.47 % to 96.95 % (Fig. 3-e). Simultaneously, the mean ESDR declined from 0.8415 to 0.8393. The deficit area of HQ is mainly concentrated in the urban living areas in the southeast, with additional deficits dispersed among the rural settlements in the mountain basin area. The proportion of the deficit area increased from 1.53 % to 3.05 %. The land use types in Yulong County are primarily composed of forest, characterized by high vegetation coverage and robust ecological resilience, fostering high habitat quality. Conversely, the central and southeastern regions are the concentrated distribution areas of cropland and built-up land.

CESDR accounts for a relatively large proportion of surplus areas (Fig. 5a), mainly distributed in mountainous areas with high altitudes. These areas have good natural and local conditions, rich natural resources, and less human disturbance, showing an ESs surplus. In contrast, the ESs deficit area is relatively small and mainly concentrated in the southeastern urban construction zone, residential areas surrounding the Lashihai wetland, and sporadically distributed basin regions. These areas experience frequent human activities, leading to robust ESs demand, but have a weak ESs supply capacity.

### 3.2 Spatial autocorrelation of ESs supply and demand

#### 3.2.1 Global spatial autocorrelation analysis of ESs supply and demand

Significance testing (p < 0.05) was conducted to analyze the output of the LISA spatial cluster map, the clustering of supply and demand for ecosystem services in 5 passed the test of significance. The global Moran's I of GP and SR in 2010 and 2020 was more significant than 0, suggesting that the two ESs types exhibited a significant positive correlation in adjacent units and a strong agglomeration effect in the whole region. In contrast, the global Moran's I of CS, WC, and HQ in 2010 and 2020 were less than 0, which indicates that these three ESs types had a significant negative correlation in adjacent units and exhibited a strong discrete effect in the whole region (Table 3).

**Table 3:** Global Moran's I of five ESs types from 2010 to 2020.

Year	Grain production	Carbon sequestration	Soil retention	Water retention	Habitat quality
2010	0.246	−0.088	0.138	−0.1134	−0.623
2020	0.234	−0.144	0.116	−0.118	−0.637

#### 3.2.2 Local spatial autocorrelation analysis of ESs supply and demand

**Fig. 4:**
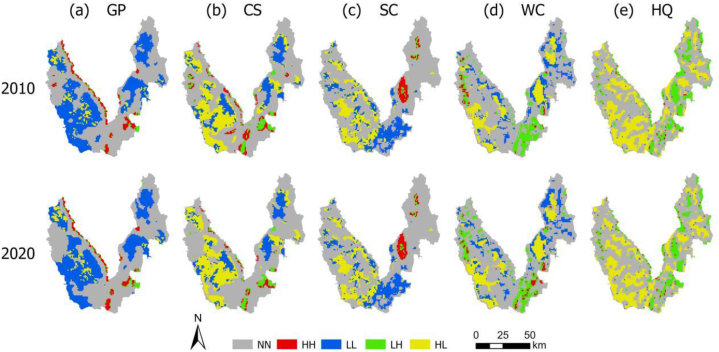
Spatial autocorrelation of the supply and demand of five ESs types from 2010 to 2020. (a) GP; (b) CS; (c) SC; (d) WC (e) HQ.

The local spatial autocorrelation results show that the supply and demand of GP are dominated by LL-type, mainly distributed in the eastern and western mountainous areas of Yulong County. The area of agglomeration change is primarily located in the central and southeastern flat basin, which has a dense population, exhibiting HH-type and LH-type areas expanding to the surrounding areas (Fig. 4-a).

**Fig. 5:**
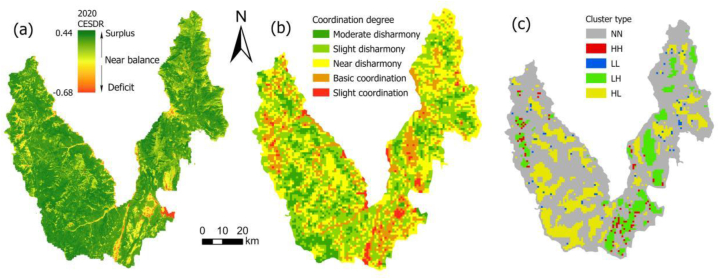
Spatial distribution of the CESDR. (a) ESs supply and demand coupling coordination degree; (b) Spatial autocorrelation of ESs supply and demand; (c) for Yulong in 2020.

The supply and demand of CS is primarily characterized by LL-type and LH-type. These are predominantly distributed in the eastern and western forest areas. Furthermore, HH-type, LL-type, LH-type, and HL-type are exhibiting a shrinking trend (Fig. 4-b).

The supply and demand of SC are primarily shaped by two distinct types: LL-type and HL-type. Specifically, the LL-type is concentrated in the central and southeastern regions, characterized by relatively flat terrain and frequent human activities. While efforts are being made to expand the LL-type areas, it is noteworthy that other agglomeration types are experiencing a declining trend (Fig. 4-c).

The supply and demand of WC are mainly LH-type and HL-type. LH-type is mainly distributed in the intermountain basin area, and HL-type is concentrated in the forest (Fig. 4-d).

The LH-type and HL-type dominate the supply and demand of HQ. Specifically, the LH-type is concentrated within contiguous forests in both the eastern and western regions. These forests exhibit high vegetation coverage, an excellent ecological environment, and minimal threats to habitat integrity. In contrast, the LH-type predominates in production and residential areas located in central and southeastern Yulong County. Unfortunately, this region experiences low vegetation coverage due to intense human activities, resulting in a situation where HQ supply is limited while demand remains high (Fig. 4-e).

The spatial agglomeration of comprehensive ESs supply and demand is primarily characterized by LH-type and HL-type, while HH-type and LL-type are scattered. The LH-type is predominantly distributed in the northeast and northwest cropland and the surrounding urban built-up areas in the southeast. Conversely, the HL-type is mainly concentrated in the eastern and western forests (Fig. 5c).

### 3.3 Analysis of CCD of comprehensive ESs supply and demand

CCD of comprehensive ESs supply and demand in Yulong County range from 0.21 to 0.68, with an average value of 0.41 (Fig. 5b). This suggests that the overall supply-demand relationship is near disharmony. CCD of comprehensive ESs supply and demand can be categorized into five types: moderate disharmony, slight disharmony, near disharmony, basic coordination, and slight coordination. Moreover, the spatial differentiation of the CCD of comprehensive ESs supply and demand is evident.

#### 3.3.1 Moderate disharmony area

The moderate disharmony area primarily occurs in forests and urban built-up areas, constituting 11 % of the study area. Forests, characterized by high vegetation coverage, provide excellent ecological quality and favorable natural conditions. In contrast, the densely populated urban built-up area has a high resource demand. However, the supply of ESs in the urban built-up area is low, resulting in a moderate disharmony between supply and demand in these regions.

#### 3.3.2 Slight disharmony area

The slight disharmony area is distributed in the transition zone between the human activity area and the forest, accounting for 35 % of the total area. In this region, vegetation coverage is sparse, soil and water conservation conditions are favorable, habitat quality is high, and human disturbance is minimal. Despite this, the balance between ESs supply and demand remains weak, with supply still exceeding demand. Overall, there exists a slight imbalance between supply and demand.

#### 3.3.3 Near disharmony area

The near disharmony area primarily occurs in rural residential areas, shrubland, and grassland, constituting 34 % of the total study area. Generally, these regions experience low economic development, have limited resource and environmental carrying capacity, and a substantial proportion of their land is dedicated to agricultural production, which significantly contributes to regional economic development. However, the ecological environment in these areas is relatively fragile, imposing considerable pressure on the coordination of ecosystem service supply and demand.

#### 3.3.4 Basic coordination area and slight coordination area

The distribution of basic coordination and slight coordination areas is consistent. These areas are mainly found in the central and southeastern flat basins with human activity, as well as in the intermountain basin areas to the east and west, along the Jinsha River coast, and within the Jade Dragon Snow Mountain Scenic Area. Collectively, these regions account for 20 % of the total area. In these areas, ESs are not only characterized by ample supply but also by significant human demand. The region boasts a flat terrain, a robust social economy, and a healthy ecological environment. Additionally, it benefits from abundant water, soil, light, and heat resources. These favorable conditions contribute to a basic coordination degree in the supply-demand relationship for ESs.

### 3.4 Ecological management zoning

**Fig. 6:**
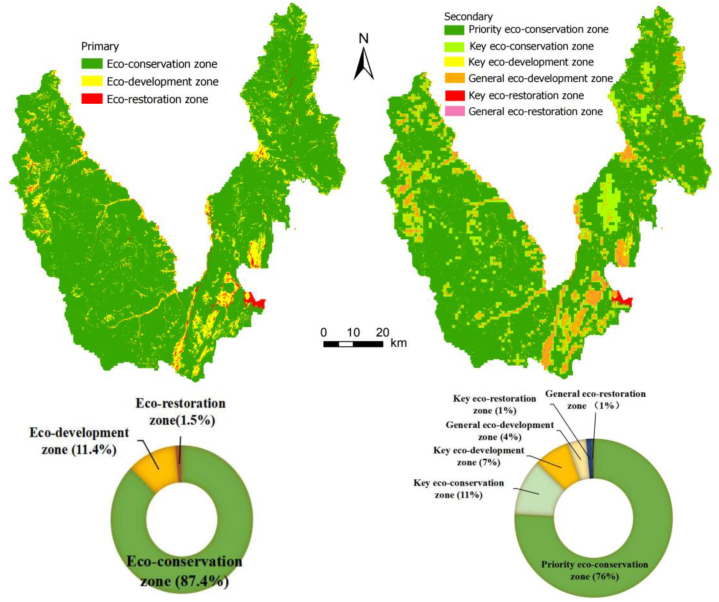
Ecological management zoning.

Considering the imbalance in CESDR, we divided Yulong County into three primary zones. We then overlay the maps of CESDR and CCD and divide the area into six secondary zones by combining them with the spatial agglomeration characteristics of comprehensive ESs supply and demand (Table 4 and Fig. 6).

(1) The eco-conservation zone covers an area of 5241.4 km^2^, constituting 87.4 % of the total study area. This zone is primarily situated in the eastern and western mountains. The zone demonstrates a surplus in CESDR, and the CCD of comprehensive ESs supply and demand exhibit moderately imbalanced to mildly imbalanced, with a predominant HL-type. The area is further divided into two secondary regions: the priority eco-conservation zone and the key eco-conservation zone. The priority eco-conservation zone serves as the core region for ecological protection, playing a vital role in safeguarding and stabilizing ecosystems. In contrast, the key eco-conservation zone is situated in the transitional area at the intersection of eco-development and eco-conservation, making it the peripheral region of the protected area. Forests occupy a substantial portion of this zone, characterized by continuous distribution and excellent integrity, which are crucial for soil and water conservation as well as maintaining habitat quality.
(2) The eco-development zone covers an area of 667.2 km^2^, constituting 11.1 % of the total area. The CESDR in this zone is nearly balanced, the CCD of comprehensive ESs supply and demand exhibiting basic to mild coordination, and primarily belongs to the LH-type. This zone is further divided into two secondary areas: the key eco-development zone and the general eco-development zone. These areas are distributed in strips, mainly in the northeast, middle east, and northwest of Yulong County, including the flat basin, the valley area along the Jinsha River, and the Lashihai Wetland. The terrain in these areas is relatively flat, with fertile soil and favorable water and heat conditions. Additionally, these areas' abundant cropland resources and excellent agricultural farming conditions are notable features.
(3) The eco-restoration zone covers an area of 90.0 km^2^, making up 1.5 % of the total area. This zone is primarily located in the county and village settlements of Yulong County, characterized by a deficit in CESDR. The ESs supply is insufficient to meet the residents' needs, resulting in an imbalance in CCD. Besides, the CESDR in this zone is primarily characterized by LH-type. This zone is further divided into two secondary zones: the key eco-restoration zone and the general eco-restoration zone. The key eco-restoration zone is mainly located in the built-up land, while the general eco-restoration zone is found in rural settlements. These zones are characterized by high levels of human activity and are prominent areas for the concentration of production and life.

**Table 4:** Ecological management zones in Yulong County.

Primary	Secondary	ES supply and demand relationships		
		ESDR	Coupling coordination	Cluster type
Eco-conservation zone	Priority eco-conservation zone	Surplus	MD, SD, ND	HL, LL, NN
	Key eco-conservation zone	Surplus	BC, SC	HH, LH, NN
Eco-development zone	Key eco-development zone	Near balance	MD, SD, ND	LH, NN
	General eco-development zone	Near balance	BC, SC	LH, HH, NN
Eco-restoration zone	Key eco-restoration zone	Deficit	MD, SD, ND	LH, NN
	General eco-restoration zone	Deficit	BC, SC	LH, HH, NN

MD moderate disharmony, SD slight disharmony, ND near disharmony, BC basic coordination, SC slight coordination, MC moderate coordination.

## 4 Discussion

### 4.1 Zoning method based on ESs supply-demand relationship

Ecological zoning based on the ESs supply-demand relationship is gaining importance and becoming more mature. However, it is worth further analyzing the matching relationship between ESs supply and demand and conducting ecological zoning research using spatial statistical methods. In this study, we selected the CESDR to reflect the region's surplus and deficit of comprehensive ESs supply and demand [54]. Additionally, we chose the CCD to reflect the actual coordination and sustainability of ESs supply and demand in the zone [55]. Utilizing local spatial autocorrelation to assist zoning can reflect the spatial continuity of ESs supply and demand [25]. This study comprehensively analyzes the spatial distribution characteristics of ESs supply-demand relationship, using both qualitative and quantitative methods to carry out ecological zoning at the grid level. As a result, we have proposed targeted management strategies.

This difference is mainly reflected in the large size of ecological protection areas and the smallest and concentrated distribution of ecological restoration areas in mountainous cities. This indicates that the unbalanced distribution of ecosystem service supply and demand in mountainous cities is closely related to topographic features [56]. Mountainous areas are high supply areas for CS, WC, and SR, while urban built-up areas in basins are high demand areas for many ecosystem services. The directions and objectives of ecological protection within different areas are formulated in response to the differences in landform types and ecological functions of the zoning [57].

### 4.2 Policy implications of ecological management zoning

The mountainous area is a crucial region for various ESs supply. However, the area has become a high demand region for ecological management and protection due to the local rocky desertification issues and the sensitivity of the mountain ecosystem. Given the imbalance between the supply and demand of ESs in Yulong County, ecological management zoning is conducted (Fig. 6). The findings of this study promote the optimal allocation of territorial space in mountainous cities, contribute to the sustainable development of local socio-ecological systems, and enrich the connotation of the theory of sustainable development. Mountainous areas account for 25 % of the global area, and they are home to 12 % of the world's population, and they are important carriers of natural ecological processes [58]. Yulong County is a typical mountainous plateau city, and the management measures proposed in this study can provide a reference for the ecological management of other mountainous cities around the world, and are of great significance for exploring the path of sustainable development in mountainous cities. The policy implications for ecological governance in different zones are as follows.

(1) The eco-conservation zone's scope is consistent with the spatial distribution of nature reserves and protected areas delineated by ecological red lines in Yulong County. This area experiences minimal disturbance from human activities and exhibits solid ecological sensitivity. Any damage to the ecosystem could result in significant natural resource loss, and the ecosystem may struggle to recover. The Jade Dragon Snow Mountain Nature Reserve plays an essential role in ecological protection in Yulong County. However, the high-intensity human activities in the scenic spot and the construction of tourism infrastructure have disrupted the ecological environment in Jade Dragon Snow Mountain. Furthermore, Jade Dragon Snow Mountain is a high-altitude rocky desertification area, and unreasonable human activities have exacerbated the process of rocky desertification. It is essential to strictly manage the ecological protection areas, limit industrial and agricultural production, and reduce infrastructure construction activities to minimize damage to the ecological environment. This strategy aids in preserving biodiversity and augmenting the supply capacity of ESs. In the eco-conservation zone, implementing ecological restoration measures, such as the Grain for Green projects and comprehensive management of rocky desertification, is recommended to enhance the ecological function of land resources [59]. Concurrently, the rational development and efficient utilization of tourism resources should be encouraged. Tourism development should be scientifically determined, and eco-tourism should be moderately developed to preserve the original ecosystem to the greatest extent possible.
(2) The eco-development zone faces significant ecological and environmental challenges, with a considerable expanse of built-up land encircling the Lashihai Wetland, which has been transformed into a tourist destination, causing severe damage to its ecological environment. In the Jinsha River Valley, extensive exploitation of cropland has resulted in severe soil erosion and agricultural non-point source pollution. To mitigate these issues, it is imperative to advocate for the construction of high-standard basic farmland, enhance agricultural infrastructure, implement farmland transformation and quality improvement initiatives, and enhance the quality and production efficiency of cropland, thereby augmenting food production and farmers' income. Furthermore, the promotion environmentally friendly agricultural technologies, which do not harm the ecological environment are essential. Developing plateau ecological agriculture will contribute to the sustainable development of agriculture in plateau areas, achieving a win-win situation between ecological environment protection and economic development [60].
(3) To address the insufficient supply of ESs in urban construction areas, it is crucial to undertake ecological restoration of urban space; this approach can enhance the ESs supply and strengthen the protection of the ecological environment [25]. It is crucial to adhere to land conservation and intensive development, improve land use efficiency, strictly control the boundary of urban development, and avoid over-exploitation. Optimizing the structure of urban green space, increasing the area of parks and leisure green spaces, and improving the availability and fairness of urban space can further meet human needs for the green landscape and promote high-quality development [61], to meet human demand for green landscapes and foster the high-quality development of urban ecological spaces.

### 4.3 Limitations and prospects

This study has a limitation in that it relies on empirical and ecological models to analyze the spatial distribution of ESs supply and demand. However, the InVEST and RUSLE models have some limitations that introduce errors in the calculation results [62]. During specific ecological planning and policy formulation, field observation should be incorporated to verify the results of ESs calculation. Additionally, the ESs demand is influenced by the preferences and cognition of various stakeholders, which complicates the calculation of the ES supply-demand relationship [63].

This study only considers five ESs types, which may only partially encompass ESs types in Yulong County. With the wealth of tourism resources, natural resources, and ecological environment, Yulong County offers significant cultural service functions. Therefore, a comprehensive and accurate assessment of the cultural service functions is needed. These ecosystem cultural services are vital for ecologically sustainable development and human well-being.

Due to limitations in data acquisition and model, this study only consideres the relationship between ESs supply and local demand within Yulong County. The interaction of ES supply and demand outside the study area and ES supply and demand within the study area is not addressed [64]. Future research could explore the spatial mobility of the ESs supply and demand and examine the flow rate, flow, and direction of ESs exchange [65]. Further studies could also be conducted on the relationship between the ESs supply and demand and human well-being, the correlation between changes in the ESs supply and demand and land use, and the dynamic simulation of the supply and demand relationship of multi-scenario ESs under different constraint conditions.

## 5 Conclusions

This study explores the relationship between ESs supply and demand and its temporal and spatial changes and then considers the spatial combination of ESDR, CCD, and agglomeration characteristics to carry out ecological management zoning, and propose targeted ecological management measures for different ecological functional zones. The primary conclusions are as follows.

(1) Aside from a marginal increase in the surplus of GP, the surplus of the other four ESs has declined. The surplus area of the CESDR is distributed in the mountainous areas with high vegetation coverage in the east and west. The areas where ESDR is close to the balance are distributed in the mountain flats, which are distributed in strips, and the deficit areas are distributed in urban construction areas and rural settlements.
(2) CS, WC, and HQ are dominated by LH-type and HL-type. Conversely, GR and SC show a negative correlation typified by LL-type and HH-type. The average CCD of comprehensive ESs supply and demand is 0.41. The CCD can be categorized into five types.
(3) Based on the differences in the spatial pattern of the imbalance between the ESs supply and demand, spatial optimization management strategies are proposed for three primary and six secondary zones.The eco-conservation zone is to strictly manage the ecological protection areas, limit industrial and agricultural production, and reduce infrastructure construction activities. The eco-development zone prioritizes improving cropland quality and ensuring food security. The eco-restoration zone focuses on enhancing ecosystem services supply and improving the structure of urban green spaces.

The results suggest that the spatial pattern of imbalance between supply and demand of ecosystem services in Yulong is related to the characteristics of mountainous urban topography. This study considers the spatial pattern of supply and demand and its partitioning results, and proposes management measures for differential ESs in different ecological zones. The proposed differential management measures can serve as a reference for governmental decision makers in ecological management. In the future, the coupling relationship between social and ecological systems in Yulong can also be studied in depth to explore more paths for sustainable development in mountainous cities and to enrich the connotation of sustainable development.

## Funding

Joint Fund of 10.13039/501100008871Yunnan Provincial Science and Technology Department and 10.13039/501100007839Yunnan University [Grant No. 2018FY001(017)], Comprehensive Investigation Project of Ecological Restoration in Alpine Canyon Area of Northwest Yunnan, China [Grant No. DD20230483], the Construction Project of Graduate Tutor Team in Yunnan Province, China [Grant No. C176230200], 10.13039/501100007839Yunnan University Postgraduate Talent Training Mode Reform Plan: The Construction Project of Joint Training Base for Postgraduate Integration Between Industry and Education at Yunnan University-Yunnan Fenglan Group, China [Grant No. CZ22622203-2022-28], the Postgraduate Innovative Research Project of 10.13039/501100007839Yunnan University, China [Grant No. 2021T008 and KC22222260].

## Data availability statement

Data associated with the study has not been deposited into a publicly available repository and data will be made available on request.

## CRediT authorship contribution statement

**Xiaobao Wang:** Writing – review & editing, Validation, Resources, Project administration, Methodology, Investigation, Funding acquisition, Formal analysis, Data curation, Conceptualization. **Xiaoqing Zhao:** Writing – review & editing, Visualization, Methodology, Investigation, Funding acquisition, Data curation, Conceptualization. **Yifei Xu:** Writing – review & editing, Resources, Project administration, Methodology, Investigation, Funding acquisition, Data curation, Conceptualization. **Yuju Ran:** Writing – review & editing, Data curation. **Xianmin Ye:** Writing – review & editing, Investigation, Data curation. **Yuqin Zhou:** Writing – review & editing, Validation. **Beihao Wu:** Writing – review & editing, Validation. **Bocheng Chu:** Writing – review & editing, Conceptualization.

## Declaration of competing interest

The authors declare that they have no known competing financial interests or personal relationships that could have appeared to influence the work reported in this paper.

## References

[1] Costanza R., d'Arge R., de Groot R. (1997). The value of the world's ecosystem services and natural capital. Nature.

[2] Daily G.C. (1997).

[3] Mathieu L., Tinch R., Provins A. (2018). Catchment management in England and Wales: the role of arguments for ecosystems and their services. Biodivers Conserv.

[4] Scholes R., Reyers B., Biggs R., Spierenburg M., Duriappah A. (2013). Multi-scale and cross-scale assessments of social–ecological systems and their ecosystem services. Curr Opin Environ Sustain.

[5] Bagstad K.J., Johnson G.W., Voigt B., Villa F. (2013). Spatial dynamics of ecosystem service flows: a comprehensive approach to quantifying actual services. Ecosyst Serv.

[6] Deng C., Liu J., Liu Y. (2021). Spatiotemporal dislocation of urbanization and ecological construction increased the ecosystem service supply and demand imbalance. J Environ Manage.

[7] Zhang L., Fu B. (2014). The progress in ecosystem services mapping: a review. Acta Ecol Sin..

[8] Ouyang X., Tang L., Wei X., Li Y. (2021). Spatial interaction between urbanization and ecosystem services in Chinese urban agglomerations. Land Use Policy.

[9] Guan Q., Hao J., Xu Y., Ren G., Kang L. (2019). Zoning of agroecological management based on the relationship between supply and demand of ecosystem services. Resour Sci.

[10] Rees W.E. (1992). Ecological footprints and appropriated carrying capacity: what urban economics leaves out. Environ Urban.

[11] Zhai T., Wang J., Jin Z., Qi Y., Fang Y., Liu J. (2020). Did improvements of ecosystem services supply-demand imbalance change environmental spatial injustices?. Ecol Indic..

[12] Millennium Ecosystem Assessment (2005).

[13] Fisher B., Polasky S., Sterner T. (2011). Conservation and human welfare: economic analysis of ecosystem services. Environ Resour Econ.

[14] Bateman I.J., Harwood A.R., Mace G.M. (2013). Bringing ecosystem services into economic decision-making: land use in the United Kingdom. Science.

[15] Yuan Y., Bai Z., Zhang J., Huang Y. (2023). Investigating the trade-offs between the supply and demand for ecosystem services for regional spatial management. J Environ Manage.

[16] Wei W., Nan S., Xie B., Liu C., Zhou J., Liu C. (2023). The spatial-temporal changes of supply-demand of ecosystem services and ecological compensation: a case study of Hexi Corridor, Northwest China. Ecol Eng.

[17] Schulp C.J.E., Lautenbach S., Verburg P.H. (2014). Quantifying and mapping ecosystem services: demand and supply of pollination in the European Union. Ecol Indic.

[18] Vargas L., Ruiz D., Gómez-Navarro C., Ramirez W., Hernandez O.L. (2023). Mapping potential surpluses, deficits, and mismatches of ecosystem services supply and demand for urban areas. Urban Ecosyst.

[19] Shi Y., Shi D., Zhou L., Fang R. (2020). Identification of ecosystem services supply and demand areas and simulation of ecosystem service flows in Shanghai. Ecol Indic.

[20] Stürck J., Poortinga A., Verburg P.H. (2014). Mapping ecosystem services: the supply and demand of flood regulation services in Europe. Ecol Indic.

[21] Sun Y., Zhao T., Cotella G., Liu Y. (2023). Ecosystem services supply and demand mismatches and effect mechanisms in the mixed landscapes context. Sci Total Environ.

[22] Zoderer B.M., Tasser E., Carver S., Tappeiner U. (2019). Stakeholder perspectives on ecosystem service supply and ecosystem service demand bundles. Ecosyst Serv.

[23] Xiang H., Zhang J., Mao D., Wang Z., Qiu Z., Yan H. (2022). Identifying spatial similarities and mismatches between supply and demand of ecosystem services for sustainable Northeast China. Ecol Indic.

[24] Xu Z., Peng J., Dong J. (2022). Spatial correlation between the changes of ecosystem service supply and demand: an ecological zoning approach. Landsc Urban Plan.

[25] Zeng J., Cui X., Chen W., Yao X. (2023). Ecological management zoning based on the supply-demand relationship of ecosystem services in China. Appl Geogr.

[26] Huang F., Zuo L., Gao J., Jiang Y., Du F., Zhang Y. (2023). Exploring the driving factors of trade-offs and synergies among ecological functional zones based on ecosystem service bundles. Ecol Indic.

[27] Zheng D., Wang Y., Hao S., Xu W., Lv L., Yu S. (2020). Spatial-temporal variation and tradeoffs/synergies analysis on multiple ecosystem services: a case study in the Three-River Headwaters region of China. Ecol Indic.

[28] Zhu J., Chang Y., Wang S. (2020). Ecological function evaluation and regionalization in Baiyangdian Wetland. Acta Ecol Sin.

[29] Yan X., Liu C., Han Z., Li X., Zhong J. (2023). Spatiotemporal assessment of ecosystem services supply–demand relationships to identify ecological management zoning in coastal city Dalian, China. Environ Sci Pollut Res.

[30] Schirpke U., Candiago S., Egarter Vigl L. (2019). Integrating supply, flow and demand to enhance the understanding of interactions among multiple ecosystem services. Sci Total Environ.

[31] Zhao X., Shi X., li Y., Li Y., Huang pei (2022). Spatio-temporal pattern and functional zoning of ecosystem services in the karst mountainous areas of southeastern Yunnan. Acta Geogr Sin.

[32] Li Z., Ma L., Chen X., Wang X., Bai J. (2023). Zoning and management of ecological restoration from the perspective of ecosystem service supply and demand: a case study of yuzhong county in Longzhong loess hilly region, China. Land.

[33] Xu Y., Zhao X., Huang P. (2024). A new framework for multi-level territorial spatial zoning management: integrating ecosystem services supply-demand balance and land use structure. J Clean Prod.

[34] Zhao X., Xu Y., Pu J. (2024). Achieving the supply-demand balance of ecosystem services through zoning regulation based on land use thresholds. Land Use Policy.

[35] Guan Q., Hao J., Ren G. (2020). Ecological indexes for the analysis of the spatial–temporal characteristics of ecosystem service supply and demand: a case study of the major grain-producing regions in Quzhou, China. Ecol Indic..

[36] Kuri F., Murwira A., Murwira K.S., Masocha M. (2014). Predicting maize yield in Zimbabwe using dry dekads derived from remotely sensed Vegetation Condition Index. Int J Appl Earth Obs Geoinformation.

[37] Liu W., Zhan J., Zhao F. (2022). The tradeoffs between food supply and demand from the perspective of ecosystem service flows: a case study in the Pearl River Delta, China. J Environ Manage.

[38] Wang C., Tang C., Fu B., Lü Y., Xiao S., Zhang J. (2022). Determining critical thresholds of ecological restoration based on ecosystem service index: a case study in the Pingjiang catchment in southern China. J Environ Manage.

[39] Mohamad R.S., Verrastro V., Bitar L.A., Roma R., Moretti M., Chami Z.A. (2016). Effect of different agricultural practices on carbon emission and carbon stock in organic and conventional olive systems. Soil Res.

[40] Renard K.G. (1997).

[41] Phinzi K., Ngetar N.S. (2019). The assessment of water-borne erosion at catchment level using GIS-based RUSLE and remote sensing: a review. Int Soil Water Conserv Res.

[42] Villamagna A.M., Angermeier P.L., Bennett E.M. (2013). Capacity, pressure, demand, and flow: a conceptual framework for analyzing ecosystem service provision and delivery. Ecol Complex.

[43] Zhu K., Cheng Y., Zhou Q., Kápolnai Z., Dávid L.D. (2023). The contributions of climate and land use/cover changes to water yield services considering geographic scale. Heliyon.

[44] Chen J., Jiang B., Bai Y., Xu X., Alatalo J.M. (2019). Quantifying ecosystem services supply and demand shortfalls and mismatches for management optimisation. Sci Total Environ.

[45] Cui F., Tang H., Zhang Q., Wang B., Dai L. (2019). Integrating ecosystem services supply and demand into optimized management at different scales: a case study in Hulunbuir, China. Ecosyst Serv.

[46] Wang B., Cheng W. (2022). Effects of land use/cover on regional habitat quality under different geomorphic types based on InVEST model. Remote Sens.

[47] Shen J., Li S., Wang H. (2023). Understanding the spatial relationships and drivers of ecosystem service supply-demand mismatches towards spatially-targeted management of social-ecological system. J Clean Prod.

[48] Zhou Y., Li J., Pu L. (2022). Quantifying ecosystem service mismatches for land use planning: spatial-temporal characteristics and novel approach—a case study in Jiangsu Province, China. Environ Sci Pollut Res.

[49] Li G., Li X., Huo L. (2023). Digital economy, spatial spillover and industrial green innovation efficiency: empirical evidence from China. Heliyon.

[50] Anselin L., Sridharan S., Gholston S. (2007). Using exploratory spatial data analysis to leverage social indicator databases: the discovery of interesting patterns. Soc Indic Res.

[51] Li W., Wang Y., Xie S., Cheng X. (2021). Coupling coordination analysis and spatiotemporal heterogeneity between urbanization and ecosystem health in Chongqing municipality, China. Sci Total Environ.

[52] Han D., Yu D., Qiu J. (2023). Assessing coupling interactions in a safe and just operating space for regional sustainability. Nat Commun.

[53] Li S., Zhao X., Pu J., Wang Q., Miao P., Tan K. (2021). Territorial space function quality evaluation and coupling coordination analysis in typical karst areas of Southwest China: a case study of Guangnan county. J Nat Resour.

[54] Li J., Geneletti D., Wang H. (2023). Understanding supply-demand mismatches in ecosystem services and interactive effects of drivers to support spatial planning in Tianjin metropolis, China. Sci Total Environ.

[55] Xin R., Skov-Petersen H., Zeng J. (2021). Identifying key areas of imbalanced supply and demand of ecosystem services at the urban agglomeration scale: a case study of the Fujian Delta in China. Sci Total Environ.

[56] Shi Y., Feng C.C., Yu Q., Guo L. (2021). Integrating supply and demand factors for estimating ecosystem services scarcity value and its response to urbanization in typical mountainous and hilly regions of south China. Sci Total Environ.

[57] Nguyen A.K., Liou Y.A., Li M.H., Tran T.A. (2016). Zoning eco-environmental vulnerability for environmental management and protection. Ecol Indic.

[58] Jansky L., Ives J.D., Furuyashiki K., Watanabe T. (2002). Global mountain research for sustainable development. Glob Environ Change.

[59] Yurui L., Xuanchang Z., Zhi C., Zhengjia L., Zhi L., Yansui L. (2021). Towards the progress of ecological restoration and economic development in China's Loess Plateau and strategy for more sustainable development. Sci Total Environ.

[60] Ma S., Qiao Y.P., Wang L.J., Zhang J.C. (2021). Terrain gradient variations in ecosystem services of different vegetation types in mountainous regions: vegetation resource conservation and sustainable development. For Ecol Manag.

[61] Lyu R., Clarke K.C., Tian X., Zhao W., Pang J., Zhang J. (2022). Land use zoning management to coordinate the supply–demand imbalance of ecosystem services: a case study in the city belt along the yellow river in Ningxia, China. Front Environ Sci.

[62] Wu Y., Tao Y., Yang G. (2019). Impact of land use change on multiple ecosystem services in the rapidly urbanizing Kunshan City of China: past trajectories and future projections. Land Use Policy.

[63] Washbourne C.L., Goddard M.A., Le Provost G., Manning D.A.C., Manning P. (2020). Trade-offs and synergies in the ecosystem service demand of urban brownfield stakeholders. Ecosyst Serv.

[64] Burkhard B., Kroll F., Nedkov S., Müller F. (2012). Mapping ecosystem service supply, demand and budgets. Ecol Indic.

[65] Wang L., Zheng H., Chen Y., Ouyang Z., Hu X. (2022). Systematic review of ecosystem services flow measurement: Main concepts, methods, applications and future directions. Ecosyst Serv..

